# Gender disparity in pediatrics: a review of the current literature

**DOI:** 10.1186/s13052-017-0437-x

**Published:** 2018-01-02

**Authors:** Paola Piccini, Carlotta Montagnani, Maurizio de Martino

**Affiliations:** 1 Post Graduate Pediatric School, University of Florence, Anna Meyer Children’s University Hospital, viale Gaetano Pieraccini 24, I-50139 Florence, Italy; 2 Pediatric Infectious Diseases Unit, Anna Meyer Children’s University Hospital, viale Gaetano Pieraccini 24, I-50139 Florence, Italy; 3 Director Post Graduate Pediatric Scool University of Florence, Director Anna Meyer University Campus, viale Gaetano Pieraccini 24, I-50139 Florence, Italy

**Keywords:** Gender, Children, Pediatrics

## Abstract

**Background:**

Gender-based medicine is an innovative branch of biomedical research and represents a new perspective for the future of health research. Many studies have been published on gender medicine in adults but very few data regarding children are available.

**Literature search and results:**

A literature search covering articles published between 1stJuly, 2006 and 1st February, 2017 and concerning children only was conducted using multiple keywords and standardized terminology in Pubmed database. The search was limited to English-language publications. All relevant articles on endocrines, neurological, psychiatric, gastrointestinal, immunological, oncological, rheumatic, pneumological disorders, infectious diseases and analgesia were evaluated and pertinent articles were included in this review. Most of the available studies on gender disparity in childhood are about endocrine and neuro-psychiatric disorders, while there are few data in other areas of medicine.

**Conclusions:**

Even if several studies on pediatric gender differences can be found on literature, few of them move forwards to analyze the reasons of the observed diversity. No data on pharmacokinetic and pharmacodynamic differences between boys and girls can be found. Hence, more efforts should be directed to investigate these topics in childhood.

## Background

Gender-based medicine is an innovative branch of biomedical research and represents a new perspective for the future of health research. The goal of gender medicine is to recognize and analyze the differences between females and males with the ultimate aim of ensuring to everyone the best possible diagnostic and therapeutic approach.

The term “gender” includes not only the differences in anatomy and physiology, but also the factors related to the environment, society, education, culture and psychology of males and females. Simply watching mortality rate of females and males in the various countries of the world, we can realize the difference in health between the genders [1]. The majority of clinical trials conducted in recent decades have mostly focused on the male gender, resulting in a bias of information on risk factors and development of diseases in the female population and, above all, a lack of pharmacokinetic and pharmacodynamic data in females. In the last decade, many studies have been published on gender medicine in adults but very few data regarding children are available, even if gender differences could be identify also in childhood. An emblematic example is represented by the situation in China, where since 1979, to put limits to overcrowding, a policy of limiting births (“one-child policy”) was adopted, allowing women to have only one child. This policy led to an increase in female infanticide and selective abortion, reaching a ratio of males and females born in 2000 to around 1.168, surpassing the 1.3 in some provinces. The mortality rate in the first year of life of girls was two times higher than of boys and the risk of death was three times higher for the second daughter. Only in October 2015, after 35 years of this policy, the Chinese government has granted to each couple the opportunity to have two sons [2, 3].

Differences between genders are reported also in the access to healthcare systems. India immunization coverage is higher in boys than in girls: in the years 2005–2006 was respectively 45.4% in males and 41.6% in females [4]. A study carried out in 2012 in Boston has documented that the access to healthcare system for children with special healthcare needs is greater for males than for females [5]. Moreover, although the rate of hospitalization in intensive care unit of boys is greater than that of girls, females have a greater mortality [6].

In 2000, a Global Alliance for Vaccines and Immunizations (GAVI) was created with the aim of creating equal access to vaccines for boys and girls living in the world’s poorest countries. In 2016, a systematic analysis of inequalities in childhood immunization conducted in 45 Gavi-supported countries found that only six countries (Lesotho, India, Burkina Faso, Gambia, Côte d’Ivoire, and Pakistan) had statistically significant differences in diphtheria-tetanus-pertussis vaccine (DTP) coverage between boys and girls. The maximum difference was in Lesotho with DTP coverage 10-percentage points higher among boys compared to girls [7].

The aim of this study is to perform a review of the scientific evidence on gender medicine in children over the past decade.

### Literature search

A literature search covering articles published between 1^st^July, 2006 and 1^st^ February, 2017 and concerning only children under the age of 18 years was conducted using multiple keywords and standardized terminology in Pubmed database, combining MeSH and free text terms for “gender” AND “children” AND “sex” AND “diseases” AND “endocrine” OR “rheumatic” OR “pain” OR “neurological” OR “gastrointestinal” OR “infectious” OR “immunological” OR “oncological” OR “pneumological”. Limits were field [Title/Abstract] and language [English]. All relevant articles were then evaluated, and pertinent articles were included in this review. The search was limited to July 2006 and not earlier in order to evaluate only the most recent studies.

## Results

After evaluation of all relevant article, 13 studies on endocrine disorders, 7 studies on neurological and psychiatric disorders, 3 studies on gastrointestinal disorders, 11 studies on infectious diseases and immunological disorders, 2 studies on oncological diseases, 1 study on rheumatic diseases, 5 studies on pneumological diseases and 5 studies on pain and analgesia were included in this review. Since the wide range of results obtained by the literature search, we cannot exclude that some important studies on gender disparity in children have been omitted by our selection.

### Gender disparity in endocrine disorders

Most of the studies obtained with our research are about endocrine disorders, in particular obesity. Song et al. have analyzed the trend in body mass index (BMI) of school-age children over the past 15 years: in females BMI remained stable, while in males increased linearly. It has been estimated that in 2020 the prevalence of obesity will be of 10.18% in males and 4.99% in females [8]. The perception of overweight is higher in women and increases with age, being adolescence a crucial period [9]. Therefore, the therapeutic-behavioral approach to overweight and obesity should be different between males and females. Munakata et al. have shown that the reduction of the hours spent in front of television is an effective weight loss method in males, while in females is more useful to promote physical exercise [10].

The different incidence of obesity is boys and girls seems to be also the consequence of nutrigenetics and nutrijenomics. For example, in Korean children, it has been recent reported that Sirtuin 1 (a longevity-associated gene) variation is associated with pediatric obesity. Anthropometrics, plasma lipid and insulin resistance profiles and nutrient intakes were analyzed with regard to 3 genotypes of SIRT1 rs7895833 (GG, GA, and AA). Among males and females, BMI and waist circumference were higher in the GA + AA group than in the GG group. Moreover, in boys, the reductions in total cholesterol and low-density lipoprotein cholesterol levels in the GG group were greater than those in the GA + AA group. In girls, the reductions in fasting blood sugar and homeostatic model assessment insulin resistance levels were greater in the GA + AA group than in the GG group [11].

Also the complications of obesity are different between females and males. In fact, as in adults, it has been shown that obese males frequently have impaired fasting blood glucose, while obese females have more often insulin resistance syndrome. This difference is not present among children under 12 years of age. Pubertal changes may contribute to the biochemical differences leading to the disparity in prediabetes [12]. Also in patients with type 1 diabetes mellitus were noted gender differences: for example, the dosage of blood C-peptide was significantly higher in females than in males, especially during puberty [13].

The response to hormonal therapies differs between males and females. Therapy with growth hormone (GH) analogs is more effective in males than in females born with a low weight for gestational age during a follow-up period of 2 years and, in the pre-pubertal age, response to treatment was significantly greater for boys versus girls also in patients with GH deficiency [14].

As it is well known, gender differences in hypothalamus–pituitary–adrenal (HPA) axis activity emerge during puberty. A systematic review recently published by van der Voorn et al. has reported that gender differences in HPA axis activity are already present in childhood: in boys aged <8 years higher salivary cortisol levels compared to girls were found and this pattern was reversed after the age of 8 years. However, gender differences in serum cortisol of boys and girls <8 years or 8–18 years were absent [15]. Moreover, in patients with congenital adrenal hyperplasia the needed dosage of hydrocortisone at puberty seems to be significantly higher in males compared to females [16].

Gender differences are also reported in thyroid pathologies. For example, in the Italian population thyroid ectopia is significantly higher in girls than in boys and moreover, girls with congenital hypothyroidism require lower doses of initial therapy in order to achieve thyroid-stimulating hormone normalization [17]. Also in Hashimoto’s thyroiditis, the most common autoimmune thyroid disease at any age, a female prevalence is reported in both children and adults, but it is more evident in the adults [18].

### Neurological and psychiatric disorders

Numerous gender differences were found in neurological and psychiatric disorders, mainly in autism spectrum disorders (ASD). It is well known that the prevalence of ASD is 15 times higher in males than in females, but also the clinical manifestations are different. Males generally have a later onset, a more severe cognitive impairment and are more frequent hyperactive, whereas females have fewer problems in communication and language development but they have social anxiety disorders [19, 20]. Numerous differences were found between males and females analyzing the characteristics of not epileptic dissociative attacks in adolescence (11–18 years) [21]. The females have more frequently atonic falls and long-term crises, while males have tonic-clonic limb movements. In males crises are frequently associated with school failure and the diagnosis of attention deficit hyperactivity disorder, while females have frequently a diagnosis of major depressive disorder [22]. Other gender differences were found in the expression of emotions. A recent review of the literature found that females express more frequently internalizing emotions (sadness, anxiety), while males show more externalizing emotions like anger [23]. Skiold et al., analyzing a cohort of infants with a gestational age less than 27 weeks, have found a better cognitive development at 30 months of age in females than in males [24].

Gender differences have also been observed in the field of neurotoxicity, especially by metals. It has been observed that males are more susceptible than females to develop neurological damage from heavy metals such as cadmium, manganese, arsenic and mercury, but the pathogenesis is unknown [25].

### Gastrointestinal diseases

Only few studies analyze the gender disparity in gastroenterological diseases. Salo et al.*,* observing 427 children less than 15 years of age underwent appendectomy, have found that females had more numerous postoperative complications, while males had often intestinal perforations [26]. It is well known that children with Crohn’s disease have height and BMI below the normal range for age. Moreover, in females the body fat levels are significantly lower than in males [27]. Yik et al. have analyzed the presence of nerve fibers containing substance P in intestinal biopsies of 76 children with chronic constipation. In fact, a low number of substance P nerve fibers seems to be associated with slow transit constipation. They have found a lower number of substance P nerve fibers in females than in males [28]*.*

### Infectious diseases and immunological disorders

Most of the studies on gender disparities in pediatric infectious diseases are related to children with human immunodeficiency virus (HIV) infection. In 2005, we reported that more females born with HIV infection, because males more often died during pregnancy [29]. More recently, in Rwanda was described that males with HIV infection are more frequently malnourished than females [30].

The females show higher plasma levels of lopinavir/ritonavir and a more rapid increase in CD4 lymphocytes after starting nevirapina than males [31]. A recent systematic review has showed that compliance to treatment is similar in both sexes. Girls with HIV infection have more difficulties in school learning than boys, although no differences in cognitive development have been identified. Moreover, males seem to develop resistance to therapy more frequently than females [32].

It is well known that the immune system is influenced by sex hormones, but it is not clear the physiological mechanism. Ghuman et al. have demonstrated a lower mortality from sepsis in adolescent females than in males. This difference has been not observed in childhood, because of the probable influence of sex hormones in the response to infections [33]. Another study had shown that the expression of inflammatory markers such as C-reactive protein, neutrophil count and erythrocyte sedimentation rate seems to be higher in females, compared to males [34]. Moreover, we showed a possible role of sex hormones in the development of tuberculosis (TB). In fact, since the TB incidence is similar in males and females during childhood, whereas during adulthood males have a higher incidence of pulmonary TB than females [35].

A recent study conducted in Tennessee has reported that rates of invasive pneumococcal diseases (IPD) were higher in male than in female subjects, particularly in children <2 years and adults 40–64 years of age, with male patients having IPD rates 1.5–2 times higher than females. The introduction of pneumococcal conjugate vaccines was associated with declines in IPD in both sexes but rates of IPD remained still significantly higher in male than in female subjects among children and adults. The mechanism for reported gender disparity in IPD rates is not clear. Males and females in this study had a similar proportion of comorbidities and so, these cannot explain the observed gender disparity. Males had higher IPD rates across all age groups but differences were more pronounced among children and young adults. Sex hormones and their concentration during the aging process may influence susceptibility to IPD and other infectious diseases. In fact, estrogen receptors were found on T cells, B cells, dendritic cells, and macrophages. However, some gender-related behaviors, such as smoking, are important drivers of IPD risk, especially among adolescents and adults but this concern is not important among young children, among whom strong gender differences have been noted [36].

Sex-differences in immune responses to viral vaccines have also been observed. Little evidence is available on this topic in infancy even though this is the age group in which most vaccines are administered. Moreover, also timing or co-administration of other vaccines can influence the immune response to vaccination. A study protocol for a meta-analysis of randomized controlled trials on this topic has been recently published but the final results will be available in 2019 [37].

It has been reported that vaccines could have gender-differential effects on mortality. A lower female-male mortality ratio (MR) after measles vaccine and an increased female-male MR after DTP has been observed in several countries (Guinea-Bissau, Senegal, Gambia, Sudan, Congo, Malawi, India, and Bangladesh). Some trials conducted in Guinea-Bissau have suggested that also inactivated polio vaccine (IPV) is associated with increased female-male MR [38].

Also timing or co-administration of vaccines could modify the female-male MR. For example, whereas *Bacillus Calmette-Guerin* vaccination co-administered with DTP-IPV was associated with lower female-male MR, subsequent DTP-IPV vaccinations were associated with an increase in female-male MR [39].

### Oncological diseases

A gender disparity in incidence and outcome of neoplastic diseases in the pediatric population is well known. Medulloblastoma, for example, has a higher incidence in males and children aged more than 3 years of age have a better prognosis [40]. Moreover, some differences in response to drugs exist. Meeske et al., for example, have reported that females treated for acute lymphoblastic leukemia have more severe long-term side effects than males [41].

### Rheumatic diseases

Rheumatic diseases typically affect more frequently females than the males. The prevalence of systemic lupus erythematosus is 9-fold higher in females than males and a different involvement of the various organs between genders has been reported. For example, at the diagnosis males rarely have alopecia, malar rash and arthralgia but these signs are more frequent in females [42].

### Pneumological diseases

Most of the studies on gender disparity in pediatric pneumological diseases are focused on asthma. There are distinct bimodal distributions for severe asthma, with an initial peak at 5 years and a second at 50 years. Males comprised the majority of individuals in the first peak in the pediatric age and women in the second in the adult age [43]. In fact, gender differences in asthma are related to age groups. The prevalence of asthma under the age of 18 is higher in boys than in girls, but in females the prevalence and severity of the disease increase with age [44]. Obesity is a major risk factor for asthma and studies reported discordant results on his influence with regard to gender [45, 46]. Gender differences are also present about cough. Majority of patients visiting clinicians for cough are adult women, who are often affected by chronic intractable cough for years. It is not known why the cough reflex becomes exaggerated in women but it seems to have the aims to protect pregnant women from gastro-esophageal reflux. Sexual differences in the cough reflex physiology are well documented by epidemiologic data from cough clinics. A higher prevalence of angiotensin-converting-enzyme inhibitor induced cough and increased excitability of peripheral and central cough pathways in women than in men are also reported by numerous studies [47]. However, no data about this point are available in children.

### Pain and analgesia

Many evidences from epidemiologic studies have demonstrated that women are at greater risk for chronic pain conditions and that postoperative and procedural pain is more severe among females than males. Both endogenous and exogenous factors may modulate pain in women and men but the mechanism underlying these sex disparities is unclear. Hormonal contributions to several pain conditions have been reported. For example, in pre-pubertal girls and boys migraine has the same prevalence but its prevalence changes to 18% for females and 6% for males after puberty. Moreover, the prevalence of one or more common pain complaints was similar among girls and boys before puberty but increased in girls after puberty [48]. However, also biopsychosocial factors seem to alter pain sensitivity differently in women and men. Depression seems to not modify pain perception in females and males, while the role of anxiety is not clear. Moreover, past individual history may have an influence in pain perception in females but not in males [49].

Few data about sex differences in chronic pain conditions among children are available. Migraine begins earlier in males than in females, with peak onset between ages of 5 and 10 years and 12and 17 years, respectively, but very few new cases of migraine are diagnosed in men during the adult age. Also the prevalence of non-migraine headache seems to be similar for school age girls and boys with increasing prevalence for girls after puberty. Moreover, girls seem to have recurrent headaches more than boys [50]. A Swedish study has compared the prevalence of headache, abdominal, and musculoskeletal pain among students. Girls were more than twice as likely as boys to suffer from headaches (17%, 8%) and abdominal pain (10%, 5%). On the contrary, there were no sex disparities for musculoskeletal pain [51].

Sex differences in response to medical and psychological treatment of chronic pain in boys and girls are unclear. Boerner et al. have recently published a meta-analysis on sex differences in the efficacy of psychological therapies for the management of chronic and recurrent pain in children and adolescents. In this study, girls reported higher depression and anxiety at pre-treatment than boys and girls with headache also reported greater pre-treatment pain severity. Treatment gains were consistent among both sexes but psychological treatment in children with non-headache pain conditions was significantly more effective in females than in males [52].

## Conclusions

Most of the available studies on gender disparity in childhood are about endocrine and neuro-psychiatric disorders, while there are few data in other areas of medicine. Even if several studies on pediatric gender differences can be found on literature, few of them move forwards to analyze the reasons of the observed diversity. Moreover, in many studies it is not possible to discriminate if differences in medical conditions are due to the gender or to socio-cultural practices in gender preferential treatment. No data on pharmacokinetic and pharmacodynamic differences between boys and girls can be found. Hence, more efforts should be directed to investigate these topics in childhood.

## Abbreviations

ASD: Autism spectrum disorders
BMI: Body mass index
DTP: Diphtheria-tetanus-pertussis vaccine
GAVI: Global Alliance for Vaccines and Immunizations
GH: Growth hormone
HIV: Human immunodeficiency virus
HPA axis: Hypothalamus–pituitary–adrenal axis
IPD: Invasive pneumococcal diseases
IPV: Inactivated polio vaccine
MR: Mortality ratio
TB: Tuberculosis
